# pH-Controlled Cerium Oxide Nanoparticle Inhibition of Both Gram-Positive and Gram-Negative Bacteria Growth

**DOI:** 10.1038/srep45859

**Published:** 2017-04-07

**Authors:** Ece Alpaslan, Benjamin M. Geilich, Hilal Yazici, Thomas J. Webster

**Affiliations:** 1 Department of Chemical Engineering, Northeastern University, Boston, MA, 02115, USA; 2 Department of Bioengineering, Northeastern University, Boston, MA, 02115, USA; 3 TUBITAK-MAM, Genetic Engineering & Biotechnology Research Institute, 41470, Gebze, Kocaeli, Turkey

## Abstract

Here, the antibacterial activity of dextran-coated nanoceria was examined against *Pseudomonas aeruginosa* and *Staphylococcus epidermidis* by varying the dose, the time of treatment, and the pH of the solution. Findings suggested that dextran-coated nanoceria particles were much more effective at killing *P. aeruginosa* and *S. epidermidis* at basic pH values (pH = 9) compared to acidic pH values (pH = 6) due to a smaller size and positive surface charge at pH 9. At pH 9, different particle concentrations did cause a delay in the growth of *P. aeruginosa*, whereas impressively *S. epidermidis* did not grow at all when treated with a 500 μg/mL nanoceria concentration for 24 hours. For both bacteria, a 2 log reduction and elevated amounts of reactive oxygen species (ROS) generation per colony were observed after 6 hours of treatment with nanoceria at pH 9 compared to untreated controls. After 6 hours of incubation with nanoceria at pH 9, *P. aeruginosa* showed drastic morphological changes as a result of cellular stress. In summary, this study provides significant evidence for the use of nanoceria (+4) for a wide range of anti-infection applications without resorting to the use of antibiotics, for which bacteria are developing a resistance towards anyway.

High rates of mortality and morbidity were a fact of life for the world’s population before the development and administration of antibiotics in the early 1940’s^1^. Arguably, antibiotics have been proven to be one of the most important medical innovations in human history, as they have reduced infectious disease-related mortality rates and healing time greatly. In the United States alone, infection-related mortality rates dropped drastically between 1900 and 1996, from 797 to 59 deaths per 100,000 people^2^. Unfortunately, the application of antibiotics may not be a viable long-term solution for infectious diseases. Over the last several decades, the excessive and improper use of antibiotics has driven the ruthless emergence of drug-resistant bacteria. Many routinely-used antibiotics are already ineffective in the clinic; some even speculate that the 21^st^ century will come to be known as the ‘post-antibiotic’ era^2^. Thus, the need for novel antibacterial agents has never been greater.

The application of nanotechnology into biology has opened up immense opportunities in many areas, including tissue engineering^3^, drug delivery^4^, diagnostics^5^, imaging^6^, and fighting bacterial infections^7^. With the emerging need for novel antimicrobial agents, nanoparticles have been proposed to treat infections as they utilize different mechanisms for killing bacteria than conventional antibiotics, making them promising candidates to overcome current issues we are facing with antibiotic drug resistant bacteria^8^. To date, nanoparticles of many different elements (such as zinc^9^, copper^10^, titanium^11^, selenium^12^, magnesium^13^, iron oxide^14^ and silver^15^) have been studied for their antimicrobial properties. It is important to note that while some of these metals, such as silver and copper, are inherently antibacterial even in their bulk form, other materials such as iron oxide^14^ only exhibit antimicrobial properties on the nanoscale^11^,^16^. So far, more than 10 different nanoparticle-based products have been commercialized for applications in bacterial diagnosis, antibiotic delivery, and medical device development^2^. However, the long-term effects associated with the use of these nanosized products are still being questioned; especially, the use of chemistries like silver, where silver has been shown to be a very effective antibacterial agent but demonstrates high toxicity to mammalian cells, or iron oxide, where iron oxide has been very efficient in killing bacteria only at high concentrations. Thus, the demand for developing effective antibacterial agents at low enough doses with minimal toxicity to mammalian cells is still badly needed.

Cerium oxide is a technologically important material due to its natural abundance in the Earth’s crust^17^ and unique physicochemical properties^18^,^19^. So far, cerium oxide has been used for a variety of applications including sensors^20^, membrane systems^21^, fuel cells^22^, mechanical polishing^23^, ultraviolet absorbance^24^, catalysis^21^, and more recently for medicine^19^,^25^ and environmental chemistry^17^. Studies concerning nanoceria’s biological applications have mostly involved the use of mammalian cells^18^,^26^,^27^,^28^. So far, nanoceria has been shown to be effective in treating cancer^5^,^29^ and inflammation^30^ by modulating reactive oxygen species levels^25^,^31^.

One of the major problems with the use of nanoparticles in suspension (and in the body) is maintaining particle size. Due to their charged surface and high surface to volume ratio, nanoparticles are in constant interaction with each other and surrounding molecules. Thus, nanoparticles tend to aggregate into clusters up to several microns. In the case of nanoceria, it has a natural tendency to aggregate as it has many oxygen vacancies on its surface^32^. Surface coatings are a common way to prevent not just aggregation, but also to enhance solubility of the particles in aqueous media. The type and amount of macromolecules coated on the particle surface have an impact on its stability as well as toxicity. Dextran is a biocompatible polysaccharide that has been commonly used in many biological applications^26^. It has been demonstrated that dextran has no effect on the redox properties of nanoceria particles^26^. However, earlier studies from our group showed that the amount of dextran has an impact on the shape, stability, and cytotoxicity of the particles. Particles with a 0.1 M dextran coating were spherical in shape with longer stability and had higher toxicity against bone cancer cells in comparison to a 0.01 M dextran coating^33^. Further investigation on their effect on healthy bone cells revealed that they had minimal toxicity against osteoblasts^5^.

While its effects on mammalian cells have been explored, relatively few studies have investigated nanoceria’s interactions with bacteria. Among these studies, some have concluded that nanoceria particles display no apparent antibacterial activity^34^,^35^. However, results from other research groups have not been as clear. For example, Thill *et al*. investigated the impact of 7 nm nanoceria particles dispersed in water on Gram-negative bacterium *Escherichia coli (E. coli*)^36^ and suggested that cerium oxide nanoparticles, which are positively-charged at physiological pH values, showed an electrostatic affinity towards the negatively-charged outer membrane of bacteria, thereby increasing the rate of particle attachment onto the cell surface. The main conclusion that can be drawn from their study is that direct spatial contact has to be made in order to provoke a cytotoxic effect of cerium oxide nanoparticles against *E. coli*.

Differences in the reported antibacterial activity of nanoceria can be ascribed to many factors such as materials used during synthesis, size, media in which the ceria was contained, and surface chemistry of the particles. In a study performed by Pelletier *et al*., a broad range of parameters, such as concentration (50–150 μg/mL), size (6–40 nm), exposure time, growth medium and pH were varied to test the growth and viability of *E. coli, Bacillus subtilis (B. subtilis*) and *Shewanella oneidensis (S. oneidensis*)^37^. Results showed a size dependent inhibition of *E. coli* and *B. subtilis*, whereas *S. oneidensis* appeared to be unaffected by the presence of the particles at all concentrations tested.

Most of the studies assessing the antibacterial activity of nanoceria particles have used uncoated particles^37^,^38^,^39^. Even though some reports claimed enhanced antibacterial activity with increasing nanoparticle concentrations, some reports suggested this toxicity is due to the aggregation of unstabilized particles and some suggested that uncoated particles interact more with the rich culture medium, thus exhibit less antibacterial activity due to their coated counterparts^40^. In a study by Cuahtecontzi-Delint *et al*., antibacterial activity of 100 nm ceria nanoparticles was enhanced by the addition of non-ionic surfactants^41^. Wang *et al*. performed a study that compared the antibacterial activity of dextran and polyacrylate coated nanoceria against *P. aeruginosa*. Ceria particles coated with dextran had a higher ability to inhibit bacteria growth, mainly due to the smaller size of the particles^42^. Similarly, in Shash *et al*.’s study, 2–4 nm dextran coated cerium oxide nanoparticles showed enhanced antibacterial properties against *E. coli*^43^.

Contrasting antibacterial responses may not only be caused by differences in treatment conditions, but also by differences in bacteria strain^44^. Importantly, variations in thickness and constituents of the cell wall in Gram-positive and Gram-negative bacteria leads to different membrane structure, surface charge density, and metabolic processes. These structural variations may also cause differences in responses to the same treatment. For instance, Pelletier *et al*. showed that nanoceria was able to inhibit the growth of *E. coli*, and *B. subtilis*, but not *S. oneidensis*, due to the fact that *S. oneidensis* is a metal-reducing bacterium^37^. Thus, the authors postulated that *S. onedensis* may be inherently more resistant to metal oxide nanoparticles than bacteria without this ability.

Overall, while reports on nanoceria’s antibacterial activity have been mixed, those studies which have shown antibacterial activity emphasize the importance of the culturing conditions. Further, the mechanism for its bacteria toxicity was shown to be dependent on whether ceria can be internalized by cells or not. For non-internalized ceria, where direct contact of the particle and cell membrane occurs, toxicity was found to be associated with reactive oxygen species (ROS) generation^45^, membrane disruption^46^ or interference with nutrient transport functions^45^. In contrast, when ceria was internalized with lysosomal injury^47^, oxidative stress^48^ was proposed to be the only mechanism of action^49^.

Based on the previous reports on nanoceria’s antibacterial activity, it is clear that nanoceria can be engineered to have antibacterial properties. Here in this study, we hypothesized that altering the pH of the solution that nanoceria is dispersed in, nanoceria size and other the physiochemical properties of the particles (like surface charge) would change to optimize an antibacterial response.

Specifically, in the current work, the pH dependent antibacterial activity of dextran-coated nanoceria was investigated. In order to achieve this objective, 5 nm nanoceria particles were synthesized, characterized in terms of size, surface charge and tested for antibacterial efficacy against two different bacteria strains: Gram-positive *Staphylococcus epidermidis (S. epidermidis*) and Gram-negative *Pseudomonas aeruginosa (P. aeruginosa*) due to their significant prevalence in implant infections. The growth of both bacteria strains was monitored after treatment with nanoceria at pH 6 and pH 9 for 24 hours, and the resulting curves were fit to a Gompertz model. Three important characteristic parameters (lag time, maximum specific growth, and the maximum cell growth) of the growth curves were calculated. Results indicated that nanoceria’s bactericidal effect is significantly more effective at basic pH values (pH 9) for both of the tested bacterial strains than at acidic pH values (pH 6). To visualize the bacteria, TEM and confocal microscopy were performed at pH 9 after 6 hours of nanoceria treatment. *P. aeruginosa* cells showed oxidative stress related elongation (also known as filamentation). This relationship between pH and bactericidal efficacy will improve the design of future nanoceria delivery strategies as an antibacterial therapy.

## Results

### Characterization of Dextran Coated Cerium Oxide Nanoparticles

**Figure 1:**
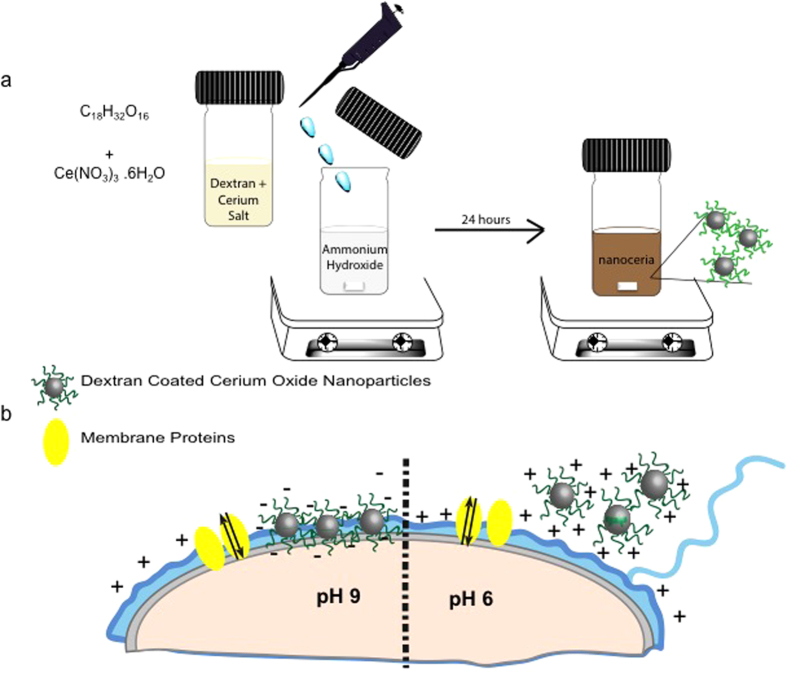
(**a**) Schematic of the nanoceria synthesis route. (**b**) Interaction between positively and negatively charged nanoceria particles and a positively charged bacteria membrane.

An aqueous solution of cerium salt and dextran was thoroughly mixed and added drop-wise into ammonium hydroxide. The color change from light yellow into dark brown within 24 hours was indicative of particle formation (Fig. 1).

**Figure 2:**
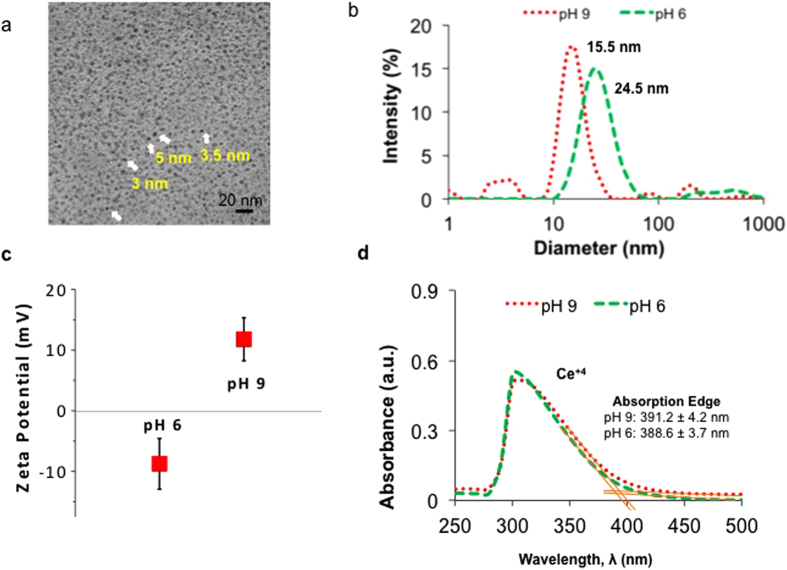
Nanoparticle characterization. (**a**) Transmission Electron Micrographs of 0.1 M dextran coated cerium oxide nanoparticles. (**b**) Hydrodynamic radius of 0.1 M dextran coated cerium oxide nanoparticles dispersed in PBS at pH 6 and pH 9. (**c**) Zeta potential of 0.1 M dextran coated cerium oxide nanoparticles dispersed in PBS at pH 6 and pH 9. (**d**) UV-visible absorption spectroscopy of 0.1 M dextran coated cerium oxide nanoparticles in TSB media at pH 6 and pH 9.

The physiochemical properties of the synthesized nanoceria particles were analyzed using a variety of techniques. TEM micrographs of the nanoceria demonstrated that particle sizes were 3–4 nm in diameter (Fig. 2a) and were spherical in shape. First, the size distribution (Fig. 2b) and surface charge (Fig. 2c) of the colloidal nanoceria particles were measured in PBS at different pH values (pH 6 and 9). While the size distributions of the particles were uniform at both pH values, the particle size at pH 9 was smaller (15.5 nm in diameter) compared to the ones at pH 6 (24.5 nm in diameter). As the pH of the environment changed from acidic (pH 6) to alkaline (pH 9), the zeta potential of the particles went from negative (−8.75 ± 4.17 mV) to positive (11.76 ± 3.54 mV).

UV-visible spectroscopy measurements were performed in order to elucidate the oxidation state of cerium oxide nanoparticles in bacteria growth media adjusted to have different pH values (Fig. 2d). While cerium can strongly absorb ultraviolet light at both oxidation states, cerium (III) absorbance occurs between the 230 nm to 260 nm range, whereas cerium (IV) absorbs light between the 300 to 400 nm range^50^. At pH 6 and pH 9 in solution, cerium oxide nanoparticles were found as Ce (IV) oxides (as a single peak was obtained at 300 nm).

### Growth of Gram-positive and Gram-negative Bacteria in the Presence of Dextran Coated Nanoceria Treatments at Different pH Values

The antibacterial activity of dextran-coated nanoceria was evaluated against a Gram-negative bacteria (*P. aeruginosa*) and a Gram-positive bacteria (*S. epidermidis*) in terms of a dose, time and pH dependent response. Bacterial experiments were performed at 250 μg/mL, and 500 μg/mL nanoparticle concentrations, at both pH 6 and 9. Lower nanoparticle concentrations (10 μg/mL, 50 μg/mL, and 100 μg/mL) were also tested under the same conditions, but not reported as there was no difference in bacterial growth in comparison to non-treated control groups.

### Antibacterial Efficacy of Nanoceria Against *Pseudomonas aeruginosa*

**Figure 3:**
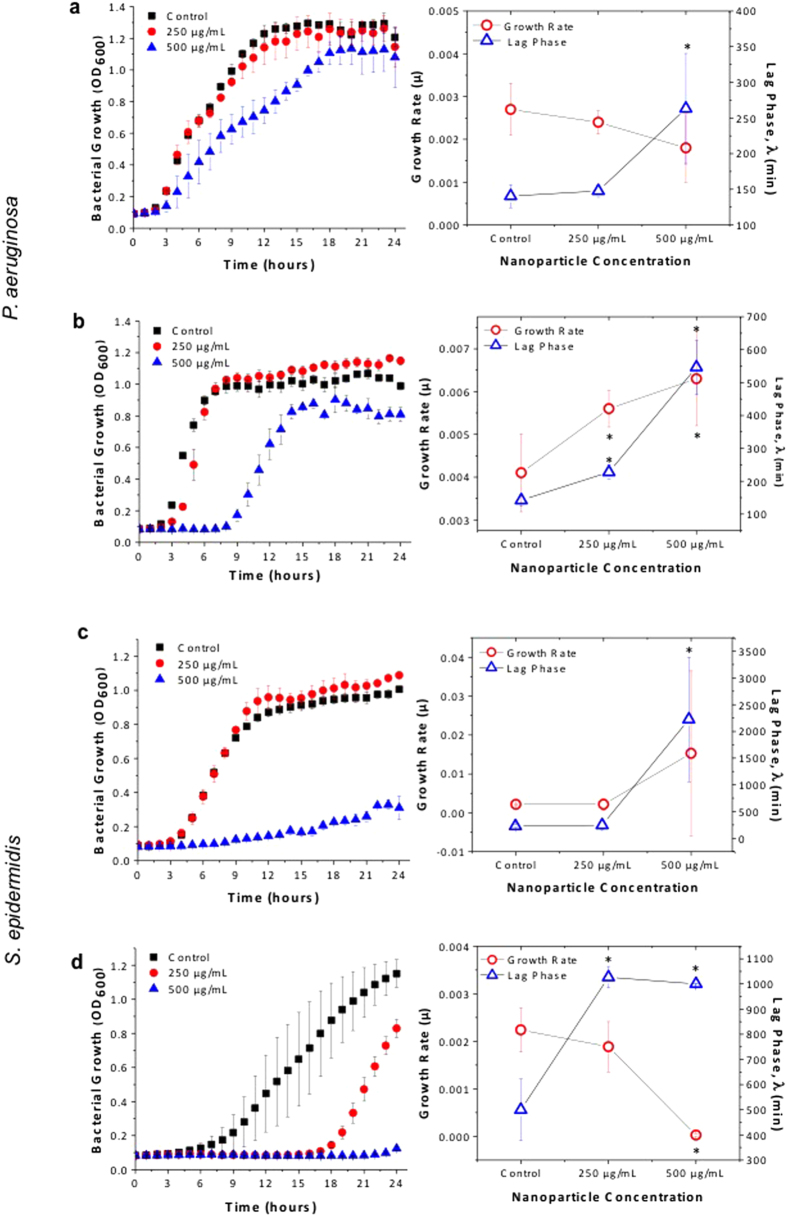
Bacterial growth inhibition and growth rate (μ) and lag phase (λ) comparison. Proliferation of 10^6^ CFU/mL of Gram-negative bacteria *P. aeruginosa* (**a,b**) and Gram-positive bacteria *S. epidermidis* (**c**,**d**) was measured over 24 hours in the presence of different concentrations of cerium oxide nanoparticles at pH 6 (**a** and **c**) and at pH 9 (**b** and **d**). Values represent the mean +/−SEM, N = 3. Corresponding Gompertz Model curve fitting parameters of each bacteria in every condition was calculated. Values represent the mean +/−SEM, N = 3 and *p < 0.05 compared to the untreated control.

At an acidic environment (pH 6), *P. aeruginosa* growth was not affected by nanoceria at 250 μg/mL (Fig. 3a). A 2 hour delay in lag time (λ) was seen in the 500 μg/mL treated samples. No significant change was observed in the calculated growth rate (μ) for both concentrations. All treatments were compared to an untreated control grown in pH 6 conditions.

At basic environments (pH 9), nanoceria was more efficient in killing *P. aeruginosa* in comparison to an acidic environment. The lag phase was significantly delayed as particle concentrations increased from 250 μg/mL to 500 μg/mL. Bacteria began to grow exponentially after 4 hours when treated with 250 μg/mL, and 9 hours when treated with 500 μg/mL. After 6 hours of culture with 500 μg/mL nanoceria at pH 9, a 2 log reduction compared to untreated controls was found (data not shown). At both pH values, the untreated control group displayed a 2 hour and 20 minute lag time, indicating that the pH of the media did not affect the initial growth of the bacteria. According to the Gompertz model curve fitting results in Fig. 3b, the calculated growth rate was significantly higher with the 250 μg/mL and 500 μg/mL treatment compared to controls (bacteria cultured at pH 9 media). Even though growth rates showed an increase with longer delays in lag phase, the nanoceria treatment at pH 9 was shown to be more effective against *P. aeruginosa*.

### Antibacterial Efficacy of Nanoceria Against *Staphylococcus epidermidis*

The growth of Gram-positive bacteria, *S. epidermidis*, was affected more by the nanoceria treatment when compared to Gram-negative *P. aeruginosa*. In an acidic environment (pH = 6), the exponential growth of the control group (bacteria cultured in pH 6 conditions) started after 4 hours. The *S. epidermidis* growth rate and lag phase was similar when treated with 250 μg/mL compared to controls (Fig. 3c). Even though bacteria growth started after the 11^th^ hour when treated at 500 μg/mL, a 7 hour delay in comparison to controls, *S. epidermidis* growth (A) was significantly lower through 24 hours due to the nanoceria treatment.

The alkaline environment affected the growth of *S. epidermidis*. The exponential growth of *S. epidermidis* started after 8 hours when bacteria were cultured in pH 9 media. Even though a significant reduction was not observed in the growth rate, the lag phase shifted for another 9 hours when treated with 250 μg/mL (Fig. 3d). Figure 3D also denotes that no bacteria growth was observed with the 500 μg/mL nanoceria treatment within 24 hours. All treatments were compared to an untreated control grown in pH 9 conditions.

### Microscopy Study of Bacteria Treated with Nanoceria

**Figure 4:**
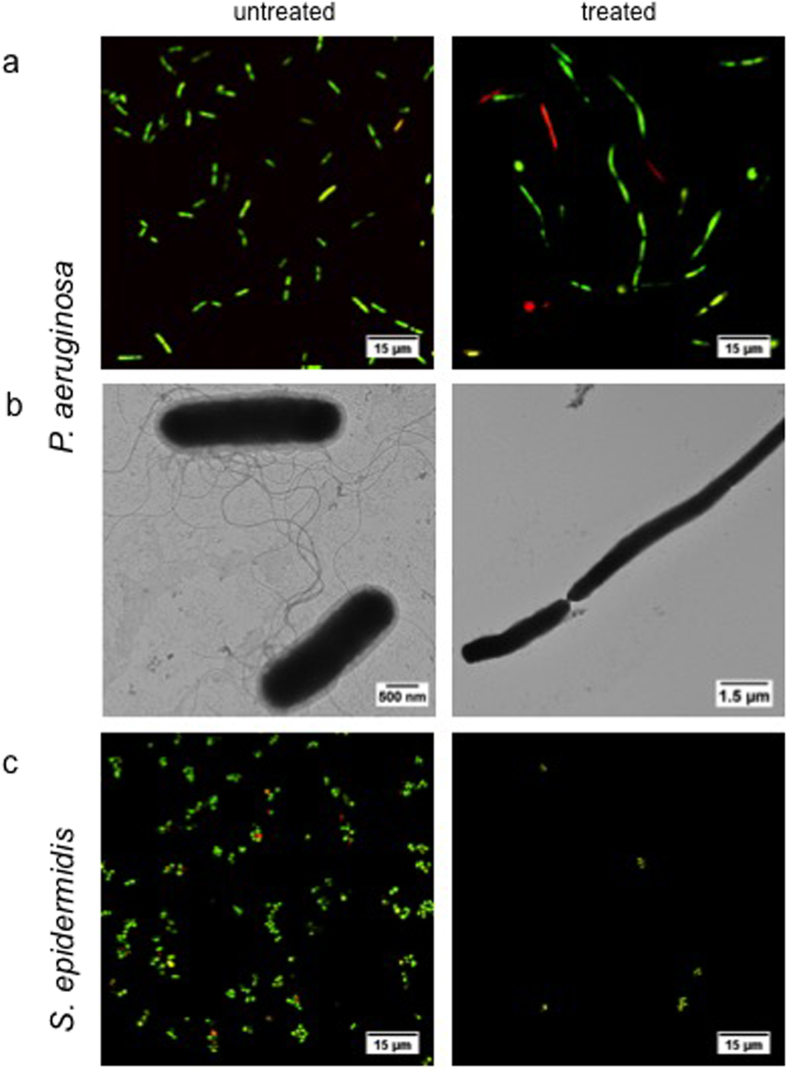
LIVE/DEAD staining and TEM imaging. The proliferation and morphology of a 10^6^ CFU/mL culture of Gram-negative bacteria *P. aeruginosa* (**a** and **b**) and Gram- positive bacteria *S. epidermidis* (**c**) was visualized after 6 hours at pH 9 both with and without nanoceria treatment.

In Fig. 4, the proliferation and the morphology of *P. aeruginosa* (Fig. 4a) and *S. epidermidis* (Fig. 4c) were visualized under confocal microscopy after 6 hours of culture with nanoceria and compared with non-treated control groups. While a reduction in the cell number was observed for *S. epidermidis* when treated with nanoceria at pH 9, both a reduction in cell number and drastic morphological changes were observed for *P. aeruginosa*. The ratio of live to dead cells did not change after nanoceria treatment of *S. epidermidis* which implies that nanoceria has a bacteriostatic effect rather than a bactericidal effect. On the other hand, *P. aeruginosa* treated with nanoceria, showed an increase in number of dead cells as well as in cell length compared to the control (bacteria cultured in a pH 9 environment) after 6 hours. This phenomenon is also known as filamentation which is a marker of cellular stress observed mostly in Gram-negative cells^51^. Filamentation of *P. aeruginosa* was also shown under the TEM in Fig. 4b in the presence of nanoceria treatment. Along with filamentation, TEM images also showed thinning and loss of the outer membrane in Gram-negative *P. aeruginosa* which may be another way of nanoceria administering its antibacterial efficacy.

TEM analysis of *P. aeruginosa* was also performed at pH 6 in order to see whether nanoceria causes any morphological changes when treated with ceria at pH 6 (images are provided in the Supporting Information as Figure S1). No morphological change was observed in Gram-negative *Pseudomonas* with nanoceria treatment at pH 6.

### ROS Generation of Nanoceria at pH 9 and pH 6

**Figure 5:**
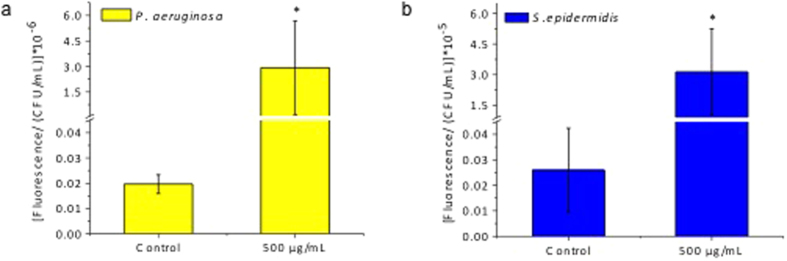
ROS generation. Reactive oxygen species generation of Gram-negative bacteria *P. aeruginosa* (**a**) and Gram- positive bacteria *S. epidermidis* (**b**) per colony after treatment with 500 μg/mL nanoceria at pH 9 for 6 hours. Values represent the mean +/−SEM, N = 3 and *p < 0.05 compared to the untreated control.

Elevated amounts of ROS cause oxidative stress within cells, eventually leading to cell death. ROS generation is known to be a prominent mechanism of cell death when cells are treated with nanoparticles. In order to see whether nanoceria treatment at pH 9 led to the generation of ROS or not, intracellular ROS generation of both Gram-negative and Gram-positive bacteria was measured (Fig. 5a and b). Since 500 μg/mL of nanoceria treatment at pH 9 was the most efficient treatment in inhibiting bacteria growth for both bacteria strains, ROS generation was detected under these conditions. Additionally, to be able to correlate these results with TEM and confocal images, incubation time was kept the same at 6 hours. As shown in Fig. 5a and b, both *P. aeruginosa* and *S. epidermidis* cells showed elevated amounts of ROS generation per colony compared to the untreated control. ROS generation per colony increased by 150 fold for *P. aeruginosa* and 120 fold for *S. epidermidis* when treated with nanoceria.

Similarly generation of ROS was also analyzed for both bacteria after 6 hours in culture with nanoceria at pH 6 (results are provided in Supporting Information as Figure S2). Results suggest that nanoceria does not cause any ROS generation for both *P. aeruginosa* and *S. epidermidis* when treated at pH 6 compared to the non-treated control.

## Discussion

The toxicity of nanoparticles can be ascribed to many factors, such as size, shape, crystallinity and surface chemistry of the particles, chemicals used in the synthesis of the particles, etc.^37^,^52^. Maintaining the size of the nanoparticles in suspension is an important challenge that is hard to achieve in the absence of a surface coating. Macromolecules are commonly used to provide steric hindrance in colloidal systems and they also enable nanoparticles to be soluble in aqueous environments; thus, in this study, dextran (a neutral, hydrophilic, biocompatible, biodegradable and branched polysaccharide) served in this capacity^53^.

0.1 M dextran coated cerium oxide nanoparticles were synthesized by an alkaline-based precipitation method. In order to confirm the size of these particles, Transmission Electron Microscopy (TEM) was performed. TEM micrographs suggested that the particles had a uniform size distribution and were sub 5 nm in spherical particle form. However, the behavior of particles may vary in colloidal form, as particles may aggregate in a kinetically driven process through the formation of clusters. Thus, it is important to analyze nanoparticles in solution. The hydrodynamic radius and size distribution of the particles were measured via DLS after dispersing them in PBS at a pH 9 and pH 6. Particles were found to be smaller (15.5 nm) in diameter at basic pH values than at acidic pH values (24 nm). The size difference under two conditions may be a factor that affects the antibacterial activity of the particles. Particles with a smaller hydrodynamic diameter at pH 9 could penetrate the thick cell membrane of bacteria more easily.

It has been reported that the antibacterial activity of cerium oxide nanoparticles is highly associated with surface charge and redox ability of cerium ions^44^,^49^. First, to elucidate what the surface charge of these particles are, and whether they change between different pH values, zeta potential measurements were performed. Results suggested that surface charge of the particles varied depending on the pH. At pH 6, nanoceria particles possessed a negative surface charge, whereas at basic pH values (pH 9), the surface charge of the particles was positive. The change in the pH can alter the surface charge of the nanoceria particles as shown here, and potentially affect the affinity of the nanoparticles to bacteria^37^. Both for Gram-positive and Gram-negative bacteria, the cell membrane is negatively charged, but the magnitude of the charge varies from strain to strain^54^. The surface charge of different bacteria studies were previously studied by Gottenbos *et al*. In this study, the zeta potential of *S. epidermidis* (HBH_2_ 102) was found to be −8 mV and *P. aeruginosa* (AK1) was found to be −7 mV^55^. An attraction between the positively charged nanoceria particles at pH 9 and the bacteria cell membrane may be one of the reasons that could explain why nanoceria particles possessed enhanced antibacterial activity. In contrast, nanoceria particles at pH 6 obtained a negative charge (−8.75 mV), which may lead to a repulsive interaction between bacteria and the nanoparticles.

Even though there are some contradictory reports on whether nanoceria is a free radical scavenger or not, its effects on the modulation of ROS levels are now well established^5^,^49^,^56^. The source of the catalytic behavior of nanoceria stems from the presence of a number of large surface defects, which are mainly oxygen deficiencies in the lattice^49^. The presence of these vacancies is responsible from the change in the local electronic stability and the valance state of the particles. Whether cerium atoms are in the +3 or in +4 oxidation state, it has a direct impact on their catalytic activity, thus, it may affect nanoceria’s antibacterial properties. Nanoceria with a higher Ce^+3^/Ce^+4^ ratio is known to have more surface oxygen vacancies, and possess more superoxide dismutase (SOD) mimetic activity, which helps to fight diseases associated with oxidative stress. In contrast, nanoceria particles with a lower Ce^+3^/Ce^+4^ ratio has more catalase mimetic activity and these particles possess more anticancer and antibacterial properties^57^. UV-Vis measurements revealed that there was a single absorption peak between 300–400 nm when nanoceria was distributed in the TSB media at pH 6 and pH 9, which corresponds to the absorbance of cerium at a +4 oxidation state. Thus, the higher ratio of Ce^+4^ in solution at both pH values may be the reason for the presently observed enhanced antibacterial properties.

The physiological and structural differences between *S. epidermidis* and *P. aeruginosa* bacteria strains resulted in different responses to the pH change in the present study without the presence of the nanoparticles. These differences may stem from the differences in bacteria membrane structure, and metabolic differences based on their respective energy generation^37^. At a neutral pH (pH 7), *P. aeruginosa* started growing after 2 hours and 20 minutes (data not shown), and as the pH of the media was changed to 6 or 9, the lag time without the particles did not change. Similar with lag phase, no significant difference was observed in the growth rate between pH 6, pH 7, and pH 9. On the other hand, for *S. epidermidis*, even though the bacteria growth started after 4 hours at pH 7 and pH 6 (data not shown), at pH 9, the bacteria growth started after 8 hours. Also, the growth rate for *S. epidermidis* treated at pH 9 media was significantly lower in comparison to the pH 6 and pH 7 media. This denotes that a basic pH had some inhibitory effect on *S. epidermidis* growth without the nanoparticles.

Another controversial aspect of ceria is whether it can be internalized by cells or not. Some previous research reported that the internalization of nanoceria does not occur in organisms like bacteria and algae due to their thick cell walls^49^. However, in human and animal cell lines and tissues, it has been reported that nanoceria can be internalized^58^,^59^. The uptake of nanoparticles usually involves two steps: (1) particle binding to the cell membrane and (2) internalization of the particles. The first step is governed by electrostatic interactions between the cell membrane and the particles. When ceria is internalized, the toxicity has been found to be associated with lysosomal injury and oxidative stress^60^. For non-internalized ceria, toxicity seems to be exerted by direct contact of nanoceria to bacteria and algae cell walls. The proposed mechanism(s) of actions are membrane disruption, ROS generation and interference with the nutrient transport functions^61^. Here, in our studies, we did not observe direct interference of particles with the bacteria cell membrane in *P. aeruginosa*. However, complete rupture of the outer membrane in Gram-negative bacteria was observed in TEM images along with a drastic increase in the size of the bacteria.

The vast majority of studies looking into antibacterial properties of nanoceria have been performed with *E. coli* as it is the most commonly studied microorganism^37^,^62^. Relatively fewer studies were performed against more clinically relevant bacteria like *P. aeruginosa*^42^,^44^. Even fewer groups studied the morphological changes in the bacteria after the treatment^39^,^45^. Here, we observed a significant morphological change in gram-negative *P. aeruginosa* after 6 hours of nanoceria treatment at pH 9. The length of *P. aeruginosa* increased drastically, and bacteria showed clumping due to particle exposure, also known as filamentation. Filamentation occurs as a result of bacteria not being able to divide, thus, generating excessive cellular stress. Elevated ROS levels were also shown at pH 9. Although never reported before for nanoceria, there are a few reports on morphological changes caused by filamentation in Gram-negative bacteria (*E. coli*) after metal oxide nanoparticle exposure (such as CdO, ZnO, and TiO_2_)^63^. Here, in our case, nanoceria had a clear effect on *P. aeruginosa* at basic pH values which resulted in cell stress leading to cell death. This study represents the first to elucidate such a mechanism of bacteria death via the use of nanoceria.

In this study, the idea was to enhance the antibacterial activity of nanoceria by altering the pH of the local environment (i.e., ion balance), thus changing the catalytic activity of the particles. A simple experimental approach was employed to compare the antibacterial activities of nanoceria at different pH values. As expected, bacteria growth decreased with increasing nanoparticle concentrations. Particles at pH 9 delayed the lag phase and reduced the total amount of bacteria for both strains more than particles at pH 6 with a reasonable presumption that once electrostatic interactions govern the proximity of particles to cells, nanoceria elevated bacteria ROS levels leading to their death. Different growth curves were observed between Gram-positive and Gram-negative bacteria. For both of the pH values, pH 6 and pH 9, Gram-positive bacteria *S. epidermidis* showed a longer delay in bacterial growth compared to the control group and *P. aeruginosa* growth curves under the same conditions. This study, thus, showed that ceria nanoparticles possess outstanding antibacterial efficacy at basic pH values (i.e., pH 9), especially for *S. epidermidis*, indicating that they can be integrated with an approach to locally increase pH to reduce bacterial infection, either with or without a medical device.

## Materials

### Syntheses of Ceria Nanoparticles

Dextran coated ceria nanoparticles were synthesized according to a modified protocol published from Perez *et al*. in 0.1 M dextran T-10 (Pharmacosmos, Holback, Denmark). Briefly, 1 mL of aqueous 1 M cerium nitrate (Sigma Aldrich, St Louis, MO) and 2 mL of dextran were mixed and the prepared solution was added dropwise to 6 mL of 30% ammonium hydroxide (Sigma Aldrich, St. Louis, MO) while stirring, and was left stirring for 24 hours at 25 °C. Upon the addition of the precursor into ammonium hydroxide, the solution turned light yellow. With the formation of stabilized dextran coated cerium oxide nanoparticles, the solution became a dark brown. After 24 hours, particles were centrifuged (4000 rpm for 30 min) to remove any debris and any large agglomerates as well as unattached dextran. The final nanoparticle solution was stored in a refrigerator at 4 °C.

Before starting any experiments, 1 mL of the ceria nanoparticle solution was dried on a hotplate to evaporate all water to calculate nanoparticle concentration (weight/mL).

### pH Adjustments

To conduct experiments at different pH values, for each nanoparticle concentration (250 and 500 μg/mL), the pH of the solution was adjusted to basic (pH 9) and acidic (pH 6) conditions. For this, after the nanoparticles were dispersed in the corresponding bacteria culture media at each concentration, an aqueous solution of 1 M to 10 M acetic acid (Sigma Aldrich, USA) was added to fix the pH values at the above-mentioned acidic values. For pH 9, an aqueous solution of 1 M sodium hydroxide (Sigma Aldrich, USA) was added dropwise. The pH of the solutions was measured with a S230 Seven Compact pH meter (Mettler, Toledo).

### Characterization of Ceria Nanoparticles

Following nanoparticle synthesis, transmission electron microscopy (TEM) (JEM 1010, JEOL) was used to observe particle size, distribution, and morphology at a 80 keV accelerated voltage. In order to determine the hydrodynamic radius, and distribution of the colloidal nanoparticles, Dynamic Light Scattering (DLS) (Malvern Zetasizer Nano ZS) measurements were performed. Particle concentration was prepared at 1000 μg/mL, dispersed in phosphate buffered saline (PBS) (Sigma Aldrich, P3813), and the pH of the solution was adjusted to either pH 6 or 9. To determine the surface charge of the particles, zeta potential measurements were performed by DLS (Malvern Zetasizer Nano ZS) and the software provided by the manufacturer.

The oxidation states were measured via UV-visible absorption spectroscopy in order to observe the changes in the oxidation states in solution depending on the pH. Particles were dispersed in Tryptic Soy Broth (TSB) (Sigma Aldrich, 22092) at a 1000 μg/mL concentration and the pH of the solution was adjusted to pH 6 and pH 9 with acetic acid (mentioned in detailed below). pH-adjusted TSB media was used as a reference to eliminate the absorption of TSB. Spectra for the cerium oxide nanoparticles were acquired using a SpectraMax M3(MT05412) at room temperature in polystyrene cuvettes.

### Bacterial Culture and Maintenance

Before bacterial experiments, for both *Pseudomonas aeruginosa* (American Type Culture Collection, 27853, Manassas, VA, USA) and *Staphylococcus epidermidis* (American Type Culture Collection, 12228, Manassas, VA, USA), a sterile 10 μL loop was used to withdraw bacteria from the prepared frozen stock, the bacteria were streaked onto a Tryptic Soy Broth (TSB) (Sigma Aldrich, 22092) Agar plate (Sigma-Aldrich, A1296), and the TSB agar plate was incubated for 20 hours, to form single bacterial colonies. For each experimental trial, a single bacterial colony of *P. aeruginosa* or *S. epidermidis* was selected and grown overnight in TSB media on a shaking incubator set at 200 rpm and 37 °C. The pH (pH 9 and 6) and nanoceria concentrations (250 μg/mL and 500 μg/mL) of the solution were adjusted as previously mentioned. The overnight bacteria suspension was adjusted by OD_600_ measurements and diluted to possess a final bacterial density of 10^6^ CFU/mL at each nanoparticle concentration. Two sets of controls were used for each experiment, one treated at the specific pH studied for each experiment, and one regular (pH 7.4) TSB media. The well plate was then incubated at 37 °C inside a spectrophotometer under static conditions (SpectraMax Paradigm, Molecular Devices, Sunnyvale, CA) with OD_600_ measurements taken every 2 minutes for 24 hours to measure the bacterial growth curves of the treatments.

### Curve Fitting

The Gompertz growth model was fitted to the experimentally-collected growth curves using a Matlab Curve Fitting Toolbox, with the Gompertz equation given as a custom function to the Curve Fitting Toolbox. Parameters determined from the experimental bacteria growth curve were maximum specific growth rate (μ), lag time (λ), and maximal growth (A).

### LIVE/DEAD Assay

A confocal microscope (Zeiss LSM 700, 63 × 1.4 N.A. Plan Apo oil objective, Zeiss, Oberkochen, Germany) with a 63X objective lens was used for imaging bacteria alone (control) and nanoceria treated (500 μg/mL) bacteria to distinguish viable cells from dead cells, and to observe any morphological changes with the help of a LIVE/DEAD BacLight Bacterial Viability Kit (L7007, Molecular probes, Invitrogen). Briefly, *P. aeruginosa* and *S. epidermidis* cells were treated with nanoceria in 5 mL cultures in a shaking incubator for 5 hours and 30 minutes at 37 °C. To adhere the cells onto the surface, Cell Tak (Sigma Aldrich, St Louis, MO) was used to coat the surface of the chambered coverglass according to the instructions from the manufacturer, and left for Cell Tak to coat on the surface of coverglass for 20 minutes at room temperature. After 20 minutes, 400 μL of the cell solution was added onto the Cell Tak treated chambered coverglass and incubated for another 30 minutes at 37 °C at static conditions to allow cells to adhere onto the surface. After the cells attached, media was aspirated from each well and replaced with 400 μL of the dye solution (1.5 μL/mL of each dye in saline solution). Cells were kept dark at room temperature for 15 minutes before the analysis.

### Nanoceria-Bacterial Interactions Observed by TEM

For TEM analysis of bacteria with nanoceria, 4 mL bacterial suspensions were exposed to the nanoceria at pH 9 and pH 6 for 6 hours in a shaking incubator at 37 °C. Cells were collected by centrifugation at 3000 rpm for 15 minutes in order to reduce the chance for artifacts, and concentrate the samples. Following centrifugation, pelleted cells were dispersed and fixed in 2.5% glutaraldehyde solution in a 0.2 M Sodium Cacodylate buffer (pH 7.4) (Electron Microscopy Sciences, 11652, Hatfield, PA) for 16 hours at 4 °C. The next day, cell morphology was visualized under the TEM.

### Determination of Reactive Oxygen Species (ROS) Generation

Intracellular ROS generation in bacteria was measured using non-fluorescent 2′,7′ Dichlorofluorescin diacetate (Sigma Aldrich, D6883). Briefly, 1 mL of bacteria cultures from the untreated control and 500 μg/mL from the treated *P. aeruginosa* and *S. epidermidis* cells were incubated at pH 9 and pH 6 for 6 hours. Then, bacteria were centrifuged at 8500 rpm for 5 minutes. A bacteria pellet was re-suspended in 30 μg/mL of a dye solution in PBS, and left in a shaking incubator at 37 °C for 45 minutes. Upon entry of DCFH_2_-DA into cells, an oxidation reaction between the non-fluorescent dye and intracellular ROS occurred, and the dye turned into a highly fluorescent compound called 2,7′ dichlorofluorescein. Cells were then centrifuged and re-suspended in 450 μl of fresh PBS, and fluorescence measured at an excitation of 485 nm, and emission of 528 nm in a black 96-well plate using SpectraMax M3 (MT05412; Molecular Devices LLC, Sunnyvale, CA).

To quantify the colony forming units (CFU) at each solution, following a 6 hour incubation, several dilutions of each solution were prepared. From each dilution, 5 * 10 μL drops were pipetted onto an agar plate and incubated for approximately 14 hours. CFUs were then counted manually. Total fluorescence was divided into Colony Forming Units (CFU)/mL in order to find the fluorescence per colony.

### Statistical Analysis

Numerical data were analyzed using standard analysis of variance (ANOVA) followed by a Student *t*-tests. All cell experiments were repeated three times, in triplicate, for all of the nanoparticle concentrations and pH values.

## Additional Information

**How to cite this article:** Alpaslan, E. *et al*. pH-Controlled Cerium Oxide Nanoparticle Inhibition of Both Gram-Positive and Gram-Negative Bacteria Growth. *Sci. Rep.* **7**, 45859; doi: 10.1038/srep45859 (2017).

**Publisher's note:** Springer Nature remains neutral with regard to jurisdictional claims in published maps and institutional affiliations.

## Supplementary Material

Supplementary Information
