# Supplementary material for: pH-Controlled Cerium Oxide Nanoparticle Inhibition of Both Gram-Positive and Gram-Negative Bacteria Growth
Source: Sci Rep. 2017 Apr 7;7:45859. doi: 10.1038/srep45859 (PMC5384081; doi:10.1038/srep45859)
Supplement: Supplementary Information [file srep45859-s1.pdf]

**Supporting Information**

# pH-Controlled Cerium Oxide Nanoparticle Inhibition of Both Gram-Positive and Gram-Negative Bacteria Growth

Ece Alpaslan[1], Benjamin M. Geilich[2], Hilal Yazici[1,3], and Thomas J. Webster[1,2,*]

*[1]Department of Chemical Engineering, Northeastern University, Boston, MA, 02115, USA,*
*[2]Department of Bioengineering, Northeastern University, Boston, MA, 02115, USA, and*
*[3]TUBITAK-MAM, Genetic Engineering & Biotechnology Research Institute, 41470, Gebze, Kocaeli, Turkey*

***Corresponding Authors:** Prof. Thomas J. Webster
Address: Department of Chemical Engineering,
Northeastern University, Boston, MA, 02115, USA
Phone: +1 617 373 6585
E-mail: th.webster@neu.edu

Supporting information consists of 2 pages and 2 figures.

Transmission electron microscopy (TEM) images of Gram-negative *Pseudomonas Aeruginosa* (*P. aeruginosa*) was performed at pH 6 after 6 hours of incubation with and without nanoceria in order to observe if there were any morphological changes in the bacteria. As shown in Figure S1, there were no significant morphological changes due to the presence of nanoceria.

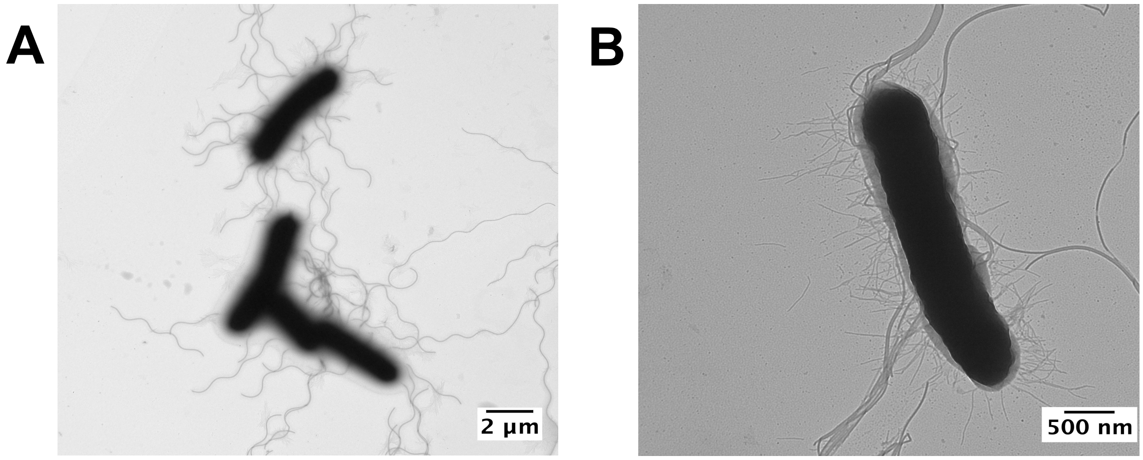


Figure S1: TEM images. The morphology of a $10^6$ CFU/mL culture of Gram-negative bacteria *P. aeruginosa* was visualized after 6 hours at pH 6 both with (A) and without (B) nanoceria treatment.

A Reactive Oxygen Species (ROS) generation assay was performed with *P. aeurginosa* and *S. epidermidis* after 6 hours in culture with nanoceria at pH 6. Results were compared to untreated control groups treated at pH 6 media. Total fluorescence was divided to number of colony forming units in 1 mL [(CFU)/ mL] and fluorescence for each single colony was reported. Results suggest that there was not any significant increase due to nanoceria treatment at pH 6 for both bacteria.

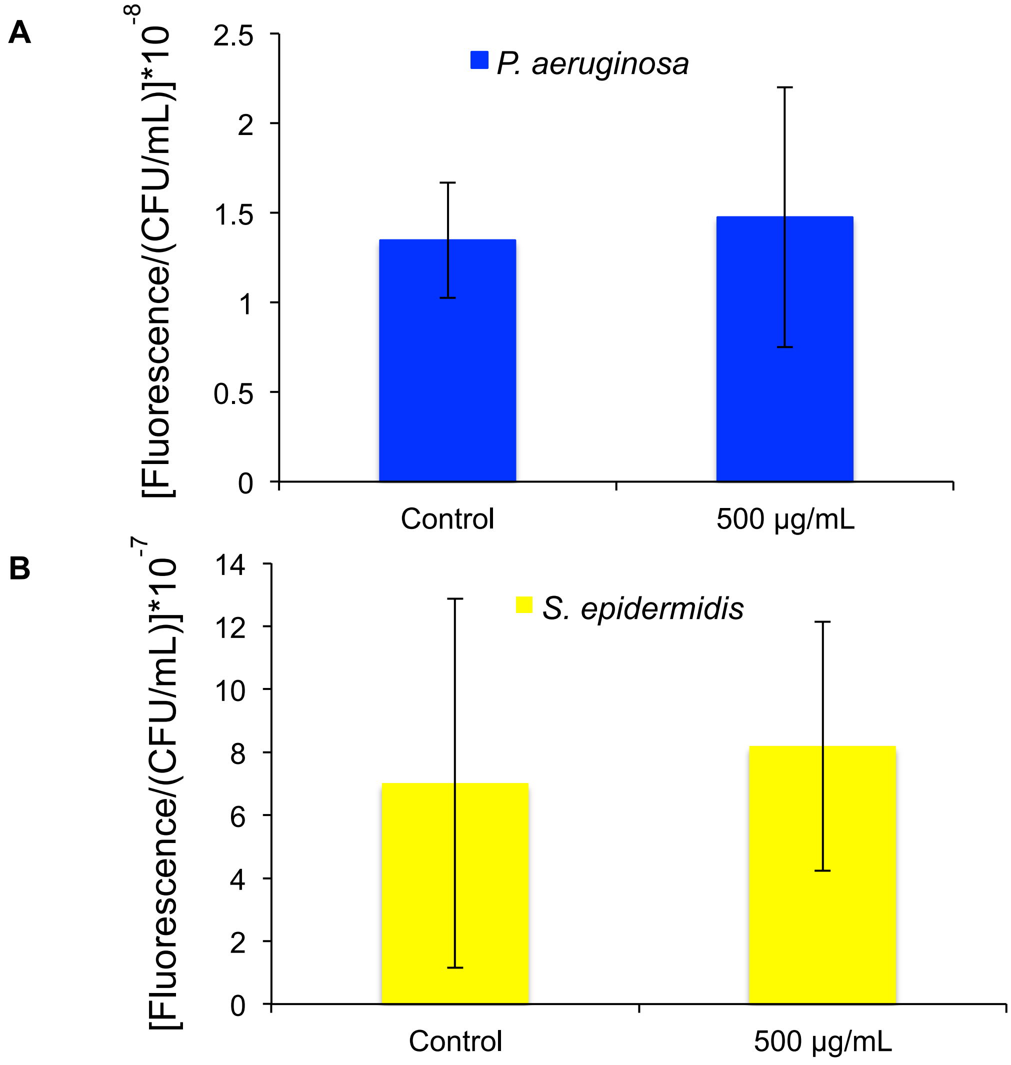


Figure S2: ROS generation. Reactive oxygen species generation of Gram-negative bacteria *P. aeruginosa* (A) and Gram- positive bacteria *S. epidermidis* per colony after treatment with 500 µg/mL nanoceria at pH 6 for 6 hours. Values represent the mean +/- SEM, N=3.
